# Case of Tetanus Cured by Etherization

**Published:** 1849-06

**Authors:** 


*Case of Tetanus cured by Etherization.* By M. Caigniet.—An infant, fourteen days old, presented the following symptoms of the disease; the jaws fixed, and visible contraction of the muscles of the face; the eyelids open and the eye immovable; the pupil a little dilated; and the lips covered with foam. The head was drawn forcibly backwards; the upper extremities were stiff, and the fingers bent; the muscles of the chest and abdomen participated in this convulsive movement, and the inferior extremities were so rigid as to permit of the infant being held by them in a horizontal position. The respiration was hurried, and the pulse small and frequent. From these symptoms M. C. considered the case to be one of tetanus, which in the absence of any external injury, he attributed to the influence of cold and moisture. He had recourse to etherization, and insensibility having been produced, the state of rigidity disappeared, the respiration bcame easier, and the pulse fell twenty-four beats in the minute. New fits came on, and were combatted by the same means. In twelve days the disease ha! altogether disappeared, but had left the child in a state of great debility. *Ibid from Bulletin de L'Acadamie Royale de Medecine de Belgique.*

